# The Role of Lifestyle and Dietary Factors in the Development of Premature Ovarian Insufficiency

**DOI:** 10.3390/antiox12081601

**Published:** 2023-08-11

**Authors:** Andrew N. Shelling, Noha Ahmed Nasef

**Affiliations:** 1 Department of Obstetrics and Gynaecology, Faculty of Medical and Health Sciences, The University of Auckland, Auckland 1142, New Zealand; a.shelling@auckland.ac.nz; 2 Centre for Cancer Research, Faculty of Medical and Health Sciences, The University of Auckland, Auckland 1142, New Zealand; 3 Riddet Research Institute, Massey University, Palmerston North 4474, New Zealand; 4 School of Food and Advanced Technology, College of Science, Massey University, Palmerston North 4474, New Zealand

**Keywords:** premature ovarian insufficiency, early menopause, ovarian ageing, diet, lifestyle, oxidative stress, infertility

## Abstract

Premature ovarian insufficiency (POI) is a condition that arises from dysfunction or early depletion of the ovarian follicle pool accompanied by an earlier-than-normal loss of fertility in young women. Oxidative stress has been suggested as an important factor in the decline of fertility in women and POI. In this review, we discuss the mechanisms of oxidative stress implicated in ovarian ageing and dysfunction in relation to POI, in particular mitochondrial dysfunction, apoptosis and inflammation. Genetic defects, autoimmunity and chemotherapy, are some of the reviewed hallmarks of POI that can lead to increased oxidative stress. Additionally, we highlight lifestyle factors, including diet, low energy availability and BMI, that can increase the risk of POI. The final section of this review discusses dietary factors associated with POI, including consumption of oily fish, mitochondria nutrient therapy, melatonin, dairy and vitamins that can be targeted as potential interventions, especially for at-risk women and in combination with personalised nutrition. Understanding the impact of lifestyle and its implications for POI and oxidative stress holds great promise in reducing the burden of this condition.

## 1. Introduction

The end of a woman’s reproductive lifespan is marked by the occurrence of menopause, defined as the last menstruation that occurs for a woman but is caused by the depletion of the ovarian reserve [1]. In the general female population, across many ethnicities and over recent human history, the average age of natural menopause has remained at about 50–52 years [1].

Premature ovarian insufficiency (POI) is a condition whereby the ovaries run out of follicles, or the follicles stop responding to normal hormonal stimulation in women under the age of 40 [2], effectively entering menopause early. However, unlike menopause, POI can be reversible. POI is also known by other terms (sometimes referred to as premature/early menopause, primary ovarian insufficiency and premature ovarian failure), and there is still limited consensus on what the actual correct term should be. However, in this review, it will be referred to as POI. POI is characterised by amenorrhoea for at least 4 months, oestrogen deficiency and two recordings of high serum follicle-stimulating hormone (FSH) at least one month apart. POI is a relatively common condition, occurring in 1% of all women and 0.1% of women under the age of 30 years [3]. As a consequence of hypoestrogenism, POI is associated with a greater risk of osteoporosis and cardiovascular disease. Women with POI have similar symptoms to natural menopause; however, these symptoms are also accompanied by an earlier loss of fertility which can be a devastating diagnosis for young women.

POI generally arises from dysfunction or early depletion of the ovarian follicle pool. In the case of ovarian dysfunction, there are sufficient ovarian follicles, but they do not respond to normal stimulation for follicles to begin folliculogenesis. This could be a result of genetic defects in signalling hormones or factors in the early folliculogenesis pathway. In a POI database from Christchurch, New Zealand, a substantial amount of diagnosed POI was found to be idiopathic [4] and likely involved a genetic contribution. POI can be “natural”, that is caused by a variety of different but identifiable factors, including chromosomal abnormalities, genetic defects and associated autoimmune diseases. However, in many cases, the cause is idiopathic or unknown. Some cases of POI are syndromic, where the POI phenotype accompanies a specific disease, such as Turner’s syndrome, Fragile X syndrome, galactosemia (due to variants in the *GALT* gene), BPES (blepharophimosis, ptosis, epicanthus inversus syndrome, due to genetic variants in *FOXL2*), and ataxiatelangiectasia (due to variants in the *ATM* gene). A recent study has shown that a wider range of genetic disorders and congenital malformation diagnoses are associated with POI, especially early-onset POI [5]. However, POI can also be “iatrogenic” such as after bilateral oophorectomy, a surgical procedure in which both ovaries are removed. In addition, chemotherapy can also lead to the early development of menopause, especially in older women with a reduced follicular reserve, which is effectively a chemical menopause [6]. POI can also arise from exposure to viral or environmental toxic agents such as smoking.

POI is a complex condition with multiple aetiologies. However, what will be highlighted in this review is the important role that oxidative stress plays in POI (Section 3). Additionally, we discuss the importance of lifestyle factors and, in particular, diet, which can both influence the risk of POI as well as can be targeted as potential interventions to reduce the burden of POI or delay the onset of POI in those predisposed.

We searched PubMed with the terms “Primary Ovarian Insufficiency”, “Premature ovarian failure”, “Early menopause”, “Ovarian ageing”, “Diet” “lifestyle”, “oxidative stress”, and “infertility” for articles published in English before May 2023. We reviewed the reference list of articles identified by this search. We focused our search strategy on systematic reviews, meta-analyses, and randomised controlled trials and selected articles based on their relevance and scientific merit.

## 2. Hallmarks of POI

Approximately 5% of all POI cases are of clear autoimmune origin (autoimmune oophoritis), and 15% of cases have a clear genetic origin [7]. Genetic defects causing dysfunction in DNA repair machinery and mitochondria and leading to dysfunction of the host’s antioxidant defences are likely to contribute to increased oxidative stress. Furthermore, an overactive immune system can lead to oxidative stress. On the other hand, iatrogenic POI, such as that induced by chemotherapy and radiotherapy, will result in increased oxidative stress due to tissue damage and apoptosis. In this section, we discuss genetic, autoimmune and chemotherapy/radiotherapy hallmarks of POI.

### 2.1. Genetic Causes of POI

The role of a genetic contribution to the aetiology of POI is supported by the observation that in about 10–30% of idiopathic cases, a first-degree relative is also affected, as well as the observation that a woman with an affected mother is six times more likely to develop POI [8]. Genetic analyses of POI patients have identified many chromosomal abnormalities, single gene mutations and genetic polymorphisms from multiple different biological pathways associated with POI development [9]. However, the genetic defects investigated thus far have been shown to contribute only a small percentage towards the development of POI [10]. The diverse aetiology of POI is in line with these findings and suggests the pathogenesis of non-syndromic POI is unlikely to be caused by a single gene or genetic defect but rather supports the view of POI being a heterogeneous genetic disease involving the interaction of multiple genetic defects and environmental factors [11]. Therefore, current research is focused on increasing the understanding of the genetic basis of idiopathic POI. This information will provide greater insight into POI pathogenesis and allow for the identification of biomarkers which may have the ability to predict early onset, providing women at risk with the opportunity to plan their families earlier and perhaps delay the onset of POI by modifying known risk factors.

As a result of recent technological advances, knowledge of the molecular basis and pathophysiology of idiopathic POI is rapidly growing with a rapidly increasing number of gene variants associated with POI. A recent review [12] highlights 107 different genes associated with POI and suggests that approximately 78% of the genes are associated with ovarian development and meiosis. New genetic technologies based on next-generation sequencing have highlighted defects in the various hormones related to folliculogenesis, reproductive hormones and mitochondria function [13]. However, there is now a growing number of genes that are implicated in DNA replication and repair, meiosis and chromosome stability that are associated with the age of menopause and POI [14]. Although the exact mechanism of how defects in DNA repair pathways and genomic instability contribute towards POI development is not yet known, it is likely that an accumulation of DNA damage and chromosomal instability in the ovary would lead to accelerated follicle atresia (which is the breakdown of the ovarian follicles, oocyte, granulosa cells and internal and external theca cells), therefore predisposing women to POI. Given that DNA damage and repair pathways are significant contributors towards POI pathogenesis, this also provides a tangible focus for potential targeted treatments [8], including lifestyle interventions such as diet and exercise. Given the already known different genetic backgrounds to the development of POI, this personalised medicine approach to match the molecular cause with potential therapeutic treatment is an important aspect to consider for the future. The role of oxidative stress and its interactions with genetic factors will be an important area of research in the future.

### 2.2. Autoimmunity and the Gut Microbiota in POI

The human ovary is commonly the target of autoimmune attacks leading to ovarian dysfunction. An estimated 20% of women with POI have an autoimmune disease comorbidity. The most common are autoimmune disorders of the thyroid, such as hypothyroidism, Hashimoto thyroiditis, and Grave’s disease [15]. The second most common autoimmune disorders are related to the adrenal glands [16,17]. Furthermore, almost 50% of women with POI are positive for at least one anti-ovarian antibody. The mechanism of autoimmune ovarian damage may be a result of abnormalities in cellular immunity [18]. This includes elevated levels of peripheral blood T-lymphocytes and the decreased number and activity of natural killer cells. Additionally, women with POI are reported to have irregularities in their levels of cytotoxic T lymphocytes and a reduced capacity for dendritic cells to aggregate with T-lymphocytes. Furthermore, it was reported that regulatory T cells (Treg) play an important role in the pathogenesis of POI [19]. In one study, it was found that there is a reduction in the number of effector Treg cells in the peripheral blood of POI patients suggesting an overactive immune system in POI [19].

The gut microbiota has an important role in the modulation and maturation of the immune system. The majority of the bacteria that colonise the gut fall within the *Bacteriodetes* and *Firmicutes* phyla, and the balance between these two phylum are important in maintaining gut homeostasis. Additionally, peptides produced from some bacterial species that colonise the gut are known to mimic molecular patterns of human tissue, resulting in a cross-reactive immune system and autoimmunity [20]. Moreover, gut dysbiosis or imbalance of the bacterial composition of the gut can activate the adaptive immunity and upregulate the innate immunity and production of autoantibodies leading to increased inflammation.

A recent study has shown that the gut microbiota was different between women with POI and healthy women [21]. Specifically, women with POI had a greater abundance of phylum *Bacteroidetes*, genera *Butyricimonas*, *Dorea*, *Lachnobacterium* and *Sutterella*. On the other hand, Phylum *Firmicutes*, genera *Bulleidia* and *Faecalibacterium* were more abundant in healthy women. The study also found that the differences in the gut microbiome of women with POI were associated with their sex hormones. In the study, oestrogen levels were significantly negatively correlated with *Bacteroidetes* and positively correlated with *Firmicutes* and *Faecalibacterium*. On the other hand, Follicle Stimulating Hormone (FSH) was significantly positively correlated with *Bacteroidetes* and negatively correlated with *Firmicutes*. Luteinising Hormone (LH) was significantly positively correlated with *Bacteroidetes*/*Firmicutes* ratio. A higher ratio of *Bacteriodetes* to *firmicutes* in women with POI indicates an imbalance in the bacterial composition and increased dysbiosis and inflammation. More recently, a study found a significantly increased abundance of genus *Eggerthella* in the faecal samples of women with POI compared to healthy women [22]. *Eggerthella* is a genus that is known to be a normal part of the microbiota but has also been linked with systemic inflammation and gastrointestinal infections [23]. Furthermore, the study found that the abundance of *Eggerthella* was not significantly different between healthy women and women with POI that were on hormone replacement therapy for more than one year [22].

Evidence suggests that there is a complex but important relationship between gut microbiota composition and sex hormones such as oestrogen [24]. Specifically, non-ovarian oestrogens in male and post-menopausal women were associated with bacterially produced β-glucuronidase enzymes [25]. β-glucuronidase is reported to block the binding of oestrogen to glucuronic acid. As a result, the levels of unconjugated/active oestrogen increase [24]. However, the influence of the gut microbiota on ovarian oestrogen and POI is not clear.

### 2.3. Iatrogenic POI: Chemotherapy and Radiotherapy

Iatrogenic POI is becoming more of an important issue as survival after treatment of malignancy through surgery, radiotherapy, and chemotherapy continues to improve [2]. Chemotherapy induces apoptosis of mature ovarian follicles, causing damage to ovaries by impairing follicular maturation or primordial follicle depletion [2]. Histological studies have shown fibrosis, vascular damage and reduced follicle numbers after chemotherapy. Chemotherapy-induced ovarian damage is dependent on the age of the patient, type of agent and the women’s ovarian reserve [26]. Older women have a higher incidence of ovarian failure after chemotherapy and an increasing likelihood of permanent infertility compared to younger women [26]. In the case of radiotherapy, irradiation of the hypothalamus, pituitary gland and pelvis is known to cause POI [27]. The application of a radiation dose of 14.3 Gray to an ovary of a woman younger than 30 years can lead to irreversible POI, while lower doses can cause reversible ovarian dysfunction [27].

Abnormalities in immunity and the gut microbiota seen in POI as well as environmental factors that induce POI, will result in increased tissue damage, inflammation and the presence of oxidative stress.

## 3. Oxidative Stress Related Mechanisms in Ovarian Ageing

Free radicals, including reactive oxygen species (ROS) and reactive nitrogen species, are natural by-products of anaerobic metabolism in the body. In normal amounts, they act as important signalling molecules that are important for physiological function. In relation to ovarian function, ROS are important in the meiotic maturation processes of oocytes [28]. However, excessive ROS leads to increased inflammation and induces apoptosis (cell death), lipid peroxidation and mitochondrial dysfunction. Additionally, the overproduction of ROS may overwhelm cellular antioxidant defences, leading to oxidative stress and premature ageing, including early ovarian ageing [29]. ROS levels in POI populations are significantly higher [30]. Oxidative stress has been suggested to be an important factor in the decline of fertility in women, including a role in enhancing early follicle loss [31]. One of the targets of oxidative stress is believed to be human granulosa cells that surround the oocyte [32]. Granulosa cells are essential for the normal development of oocytes since they produce steroidal hormones and growth factors that support the cells. Given that granulosa cells play a central role in follicle development, growth, and follicular atresia, it stands to reason that loss of granulosa cell function may be an important component of early loss of ovarian function.

### 3.1. Mitochondrial Dysfunction

The maturation of ovarian follicles requires orchestrated crosstalk between the oocytes and the surrounding granulosa cells. Excessive production of ROS leads to increased apoptosis of the granulosa cells and mitochondrial dysfunction [28]. Mitochondrial dysfunction leads to further production of ROS, which results in further damage to ovarian cells.

Mitochondria are the powerhouse of the cell. The main function of mitochondria is to produce energy through adenosine triphosphate (ATP) by oxidative phosphorylation (OXPHOS). Oocytes require a lot of energy which is supplied by the mitochondria, to allow for oocyte maturation, fertilisation, and to maintain embryogenesis [28]. One of the main by-products of OXPHOS is the generation of ROS, making the mitochondria the primary source of intracellular ROS production. Mitochondrial dysfunction has been shown to disrupt the bidirectional interaction between the oocytes and granulosa cells which leads to disruption of cell growth and development [29]. Furthermore, mitochondrial dysfunction that causes increased ROS has been reported to disrupt meiotic spindles in oocytes, which compromises their function and quality [33,34]. Mitochondria are unique in that they contain their own DNA, known as mtDNA. Increased ROS results in mtDNA damage and dysfunction. Patients with POI were shown to have significantly decreased mtDNA compared to healthy fertile women [35]. Additionally, several mitochondrial-related gene mutations have been identified that give rise to either isolated POI or POI as part of a syndrome, including *MRPS22*, *POLG*, *TWNK*, *LARS2*, *HARS2*, *AARS2*, *CLPP*, and *LRPPRC* [7].

### 3.2. ROS Apoptosis and Inflammation

Apoptosis is programmed cell death that is used by the body to get rid of unwanted or defective cells. Apoptosis is a regulatory process in the ovaries for the maintenance of healthy follicles and eliminating cells without triggering an inflammatory response [36]. However, apoptosis is also considered one of the most important mechanisms in ovarian ageing [28]. One of the pathogenic mechanisms in POI is the extensive follicular atresia through apoptosis of the granulosa cells [37]. Follicular atresia refers to the fate of any follicles that are not destined for ovulation and is mainly via the process of apoptosis. Follicular atresia begins before birth and continues throughout a female’s life until menopause [36]. Throughout the reproductive period, women will lose around 250,000 follicles, with an estimated rate of about 1000 follicles per month through atresia, leaving only about 400 follicles progressing to ovulation [36]. Apoptosis targets mainly granulosa cells in adult life, and increased granulosa cell death can lead to defective follicle development and suppress the growth of the oocyte [38]. Furthermore, apoptosis of granulosa cells is associated with reduced success rates of conception and pregnancy through in vitro fertilisation embryo programs [39,40].

**Figure 1:**
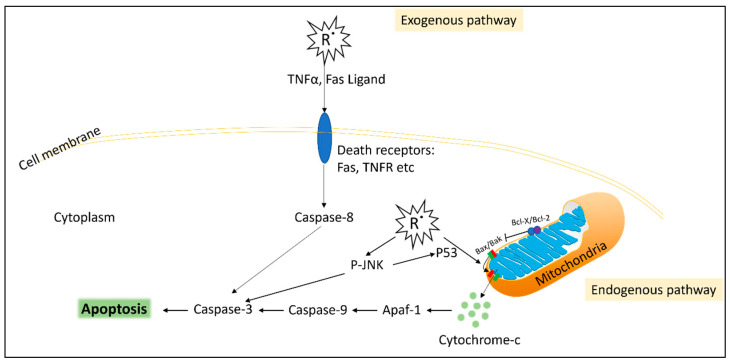
Simplified diagram of reactive oxygen species (R˙)-induced apoptosis. Exogenous pathway: Free radicals induce death receptors such as the TNF receptor (TNFR) or FAS receptor, as well as inducing the production of their ligands TNFα and FasL (respectively) from neighbouring cells. The death receptors stimulate caspase-8, which activates apoptosis. Intrinsic pathway: cellular free radicals, such as H_2_O_2_, result in mitochondrial dysfunction via the Bax/Bak leaking of cytochrome-c from the mitochondria, which activates apoptosis via the caspase complex. Cellular free radicals also result in the phosphorylation of JNK (P-JNK) and P53, which leads to further activation of apoptosis.

There is a large body of evidence attributing oxidative stress as a major contributing factor in triggering ovarian apoptosis (Figure 1) [28,41]. Within the ovarian tissue, excess ROS has been shown to activate apoptosis by the FasL pathway and recruitment of caspase-8 in mice [42]. Additionally, apoptosis can be induced endogenously by ROS disrupting the mitochondria. Mitochondrial dysfunction results in the release of cytochrome C which activates caspase-9/3 and upregulation of the Bax/Bcl-2 ratio leading to the initiation of apoptosis [28,41]. Additionally, excess ROS has been shown to activate the pro-inflammatory cytokine TNFα, which activates apoptosis via the caspase-8 pathway [28,41]. A study also identified that ROS activates the apoptosis pathway via increasing phosphorylation of JNK and P53 in a granulosa cell line induced by H_2_O_2_ pro-oxidant [32]. In that study, treatment with an antioxidant N-acetyl-l-cysteine prevented the phosphorylation of p53 and JNK [32]. Furthermore, cell debris from apoptosis leads to cell-free DNA fragments in the ovarian environment, which stimulates more ROS, further exacerbating apoptosis [43]. Sustained levels of ROS also induce pathological necrosis and necroptosis cell death in the ovaries [44].

### 3.3. Oestrogen and Oxidative Stress

**Figure 2:**
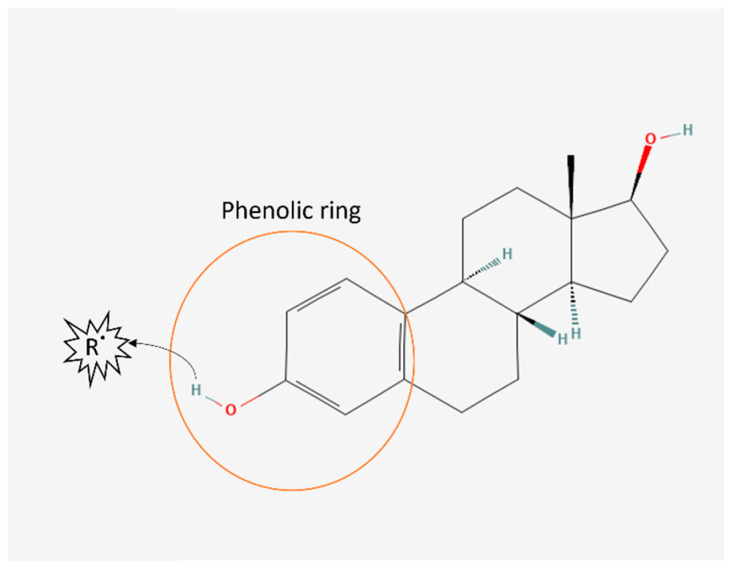
Oestrogen antioxidant structure. Oestrogen chemical structure [52] contains a phenolic ring capable of scavenging free radicals (R˙) by transferring a hydrogen atom to the free radical.

Oestrogen is well known to possess antioxidant properties. They are a ROS-scavenging hormone due to possessing a phenolic ring (Figure 2). However, the amount of circulating oestrogen in women is thought to be too low to directly elicit significant antioxidant effects [45]. Oestrogen can also upregulate antioxidant enzyme activity, such as glutathione peroxidase and manganese-superoxide dismutase [45]. Experimental and animal studies have supported oestrogen’s antioxidant properties [46,47,48,49]. All three phenolic oestorgens found in mammals (oestrone, oestradiol and oestriol) were shown to have 2.5 times higher total antioxidant capacity compared to vitamin C in vitro [46]. Other studies in female mice [47] and pigs [48] administered with oestradiol had significantly reduced lipid peroxidation. However, the antioxidant effects of oestrogen in humans are less clear. In the BioCycle study monitoring reproductive hormonal cycles in healthy premenopausal women, oestrogen levels was positively associated with F2-isoprostanes, a biomarker of oxidative stress [50]. In the same study, serum antioxidants ascorbic acid, tocopherol and lutein were positively associated with serum oestrogen [51]. These studies suggest that oestrogen plays an important role in oxidative stress and that disturbances in oestrogen levels can impact fertility.

## 4. Risk Factors for Primary Ovarian Insufficiency

### 4.1. Diet

A number of observational studies reported a link between increased intake of vegetarian-type food and earlier onset of menopause. A systemic review identified that increased consumption of yellow and green vegetables was associated with earlier onset of natural menopause [53]. Additionally, observational studies found associations between the consumption of soy products [54] and a trend for increased consumption of cereal, fibre and vegetables, with increased risk of earlier onset of menopause [55]. Furthermore, in a cross-sectional study, it was reported that not being vegetarian was associated with later age of natural menopause onset [56]. A more recent study reported that there was no association between consuming a healthy plant-based diet and early menopause; however, there may be an increased risk with an unhealthy plant-based diet [57]. Similarly, a large long-term cohort study (UK Women’s Cohort Study) followed 14,172 participants over 4 years and found that the intake of refined pasta and rice was associated with earlier natural menopause, whereas intake of fresh legumes was inversely associated with the onset of natural menopause [58]. Additionally, studies found high intake of vegetable protein [59] and higher fruit intake [53] was associated with a delay in natural menopause. The reports from observational studies around the consumption of vegetarian or plant-based diets and food have been conflicting [19]. However, studies on the mechanism of action can shed some light on some of these reports.

Plant-based diets and food, while high in beneficial polyphenols that have antioxidant properties, are also a source of phytoestrogens (as reviewed in [60]). Phytoestrogens are non-steroidal polyphenolic compounds naturally found in plants, including legumes, soybeans, beans, nuts, cereals, flax seeds, sesame seeds, hops and other plants. These compounds are similar to mammalian oestrogens, with effects exerted on the reproductive system. They exert their oestrogenic effects by binding oestrogen receptors, acting as agonists, partial agonists and antagonists. There is a good deal of literature on the benefits of phytoestrogens on reducing atherosclerosis, osteoporosis, angiogenesis, type 2 diabetes and vasomotor effects (hot flushes and night sweats) at menopause and their antioxidant and anti-inflammatory properties. However, animal studies have shown that phytoestrogens can affect reproduction [60]. Temporary and permanent infertility was seen in ewes and cattle consuming clover (a source of high concentrations of phytoestrogens). Furthermore, a diet high in phytoestrogens was a major contributor to decreased fertility in the cheetah population. In humans, data suggest that food rich in phytoestrogen soy isoflavone may suppress oestrogen and progesterone concentration and the preovulationary surge of LH and FSH and delay menstruation [60]. While the effect of a higher intake of phytoestrogens in leading to earlier onset of permanent menopause is unsupported, no studies have looked specifically at the effects of these compounds and the risk of POI and temporary infertility.

### 4.2. High Fat Diet

Increased consumption of fat can lead to unfavourable physiological conditions in the body [61,62]. To accommodate surplus fat consumption, adipocytes start to undergo excessive hypertrophy that leads to increased adipose tissue inflammation, oxidative stress and endoplasmic reticulum stress [61]. Additionally, chronic overnutrition causes intracellular lipid accumulation in other tissues leading to lipotoxicity, insulin resistance, cellular dysfunction and apoptosis in multiple organs [61]. High levels of intracellular fatty acids can impact the mitochondrial membrane structure causing mitochondrial dysfunction.

In the ovaries of mice fed a high-fat diet, oocytes contained high levels of lipids [63]. Furthermore, obese women are reported to have increased levels of triglycerides in follicular fluid [64]. In obese women, failure to achieve pregnancy was only seen when autologous oocytes were used but not with donor oocytes [65], suggesting obesity impairs oocyte quality, possibly due to elevated lipid content. In a mouse study, a high-fat diet resulted in oocytes with delayed maturation [66].

Few human studies have specifically focused on the effects of dietary fat intake and reproduction; however, it is likely to be associated with altered reproductive function [62]. In one study with healthy premenopausal women, the menstrual cycle tended to be longer after consuming a 40% fat diet compared to when they consumed a 20% fat diet [67]. Another study with South African women showed that when the women were switched to a Western-style diet by introducing meat, which raised their fat intake by 5% for two months, it resulted in increased FSH levels, decreased oestrogen, and increased length of the follicular phase of their cycle [68]. A high-fat diet may therefore contribute to an increased risk of POI.

### 4.3. BMI

The link between BMI and ovarian failure is unclear and inconsistent, with few research studies looking at the relationship. Body mass index (BMI) is a measure of body fat based on an individual’s weight and height and is often used as a surrogate measure of adiposity. High BMI has been associated with a range of health conditions, including reproductive problems and infertility [69]. However, there is only limited evidence for a link between high BMI and age of menopause and less evidence for a link to POI. Low BMI can also lead to reproductive problems, such as Relative Energy Deficiency in Sport (RED-S), which may result in delayed onset of puberty, amenorrhea, and other menstrual abnormalities [69]. While poor nutrition can potentially impact reproductive health, there is not strong evidence to support a direct causal relationship between malnutrition and early menopause in women. However, women who are severely malnourished will experience early menopause [70,71] or amenorrhea in young women [72].

Various studies have found a modest association between being underweight and earlier age at menopause [73] and being overweight and later age at menopause [74]. Given the association of obesity with inflammation and oxidative stress and the likely impact on ovarian function, this does sound counter-intuitive. In addition, many of the studies are not adequately controlled for the effect of smoking on body weight; smoking is known to have a significant impact on the age of menopause [74].

One explanation for the link between BMI and the age of menopause may be the production of oestrone in the adipose tissue in overweight and obese women [75]. In adipose tissue, circulating androstenedione produced by both the ovary and adrenal glands is converted into oestrone, a weak oestrogen, by the enzyme aromatase [75]. Aromatase expression and activity in adipose tissue are known to increase as a function of body weight and age [75]. Oestrone will contribute to the amount of total oestrogen a woman will have and is positively related to body weight. It is believed that this additional oestrogen will contribute to the delay in menopause.

Another possible link between BMI and the age of menopause is the production of leptin in adipose tissue. Leptin has a role in communicating information on the body’s energy stores to the hypothalamus and plays a role in the initiation of puberty and regulation of normal reproductive function. A study has shown that early menopause is associated with low leptin levels [76]; however, there is clearly a need for more studies in this area to confirm the relationship between leptin and the age of menopause.

A clear mechanistic link between being overweight and reduced reproductive function appears to exist. It is known that being overweight can increase oxidative stress in the body through a number of potential mechanisms, including increased production of ROS in adipose tissue [77,78]. Similarly, obesity is associated with chronic low-grade inflammation in the body [79]. Adipose tissue is an important endocrine organ that produces adipokines, and it is believed that they contribute to a state of inflammation. Lifestyle modifications, such as a healthy diet, engaging in regular physical activity, and weight loss, can help reduce inflammation and mitigate the adverse effects of obesity on inflammation-related health conditions.

The role of the gut microbiome is also central to reproductive health. Obesity does alter the composition of the gut bacteria, which in turn will lead to an inflammatory response. The altered gut bacteria, or dysbiosis, may have a number of effects, including imbalances in inflammatory and immune responses. However, it remains controversial whether the altered gut microbiota is a risk factor for or a consequence of obesity.

### 4.4. Exercise

It is accepted that regular exercise is recommended as an important part of a healthy lifestyle. However, markers of oxidative stress and elevated cortisol are produced during high-intensity exercise [80]. Furthermore, the rapid introduction of heavy exercise, particularly in combination with low energy availability (RED-S), is associated with ovulatory and menstrual cycle disturbances [81,82]. However, these disturbances, which include increased cortisol, decreased progesterone and shortened luteal phase, appear to be subtle [83]. Intense exercise may lead to amenorrhea (defined as no menstrual cycle for 3 or more months) in women who exercise vigorously if their body weight reduces below certain critical levels in addition to suffering from other stressors such as illness, calorie restriction or inappropriate eating habits or psychological stress [82,84]. In contrast, exercise appears to restore ovulation in overweight and obese women [81].

While exercise is widely recognised to be beneficial for overall health and well-being, there does not seem to be any direct evidence that it has any role in the development of POI. Yet, there is some limited evidence to suggest that exercise may be related to the age at which a woman reaches menopause; however, the relationship appears to be complex, and many other factors are likely to be involved [84]. While some studies have found that women who engage in regular physical activity tend to experience menopause at a later age compared to women who are sedentary [84], other studies have found no relationship [85].

### 4.5. Other Lifestyle Factors

#### 4.5.1. Smoking

Studies have implicated cigarette toxins in subfertility, endocrine disorders, earlier onset of menopause and POI [86]. The early onset of menopause associated with smoking suggests an association with early follicular depletion. A meta-analysis [87] showed that the risk of infertility in women smokers versus non-smokers was significantly increased (odds ratio 1.60; 95% CI 1.34–1.91). Smoking strongly damages the quality of oocytes and results in a reduction of ovarian reserve in women [88].

#### 4.5.2. Psychological Stress

Anecdotally, many women with POI often link the onset of ovarian failure to a period of prolonged pressure or stress, but evidence to support this relationship has been limited, and exact causal mechanisms are even less clear.

Stress can be defined as a feeling of emotional strain and pressure. Some levels of stress may be seen as being beneficial, and there is some evidence that it can actually improve performance in a number of different environment settings. However, ongoing or excessive amounts of stress can lead to health problems, such as depression and strokes. When an individual experiences a stressful situation, the body responds by activating the “fight or flight” response, the hypothalamic-pituitary-adrenal axis and sympathetic nervous system are activated, and stress hormones such as cortisol, adrenaline and nor-adrenaline are released. These hormones may help to prepare the body to deal with stressful situations. Psychological stress is where an individual may encounter emotional strain and pressure in a situation that exceeds their ability to cope effectively with it. Over time, excessive psychological stress can have negative effects on a person’s overall well-being and mental health, leading to a range of physical, emotional, and behavioural symptoms.

In the same way, excessive psychological stress can influence reproductive health, disrupting the balance of hormones in the body as part of the hypothalamic-pituitary-gonadal axis, potentially affecting the menstrual cycle and folliculogenesis. This has sometimes been referred to as functional hypothalamic amenorrhoea, with multiple risk factors and links to stress-related, weight-related, and exercise-related factors [89].

Few studies have shown a link between POI and stress, although the occurrence of POI has been associated with depression, anxiety and other negative emotions [90,91]. In addition, animal studies of chronic unpredictable mild stress showed that stress could induce decreased ovarian reserve in female rats and depression-type behaviours [92]. Clearly, there does seem to be a link between long-term psychological stress, but further studies are required to evaluate the interaction between psychological stress and ovarian function to understand the mechanisms of stress-induced dysfunction.

#### 4.5.3. Ageing

Reduced oocyte quantity and quality are associated with maternal ageing. There is increasing evidence that DNA damage increases and repair mechanisms become increasingly inefficient as women age [93]. Additionally, aneuploidy or an unbalanced chromosomal complement in oocytes is associated with increasing female age, as evidenced by an increased rate of miscarriage [94,95]. A comprehensive study reported that aneuploidy occurs in 20% of the oocytes of women from 35 years and increases to almost 60% of the oocytes in women over 43 years of age [36]. Some of the reasons behind the increased aneuploidy in ageing include increased errors occurring during meiotic cell division in older oocytes and reduced stringency in checkpoints during oocyte meiotic activation [96]. Oocyte quality is also affected by increased mitochondrial dysfunction as a function of ageing. In one study, mitochondrial activity, represented by ATP production and metabolic activity, diminishes with maternal age during oocyte maturation [97]. As a consequence of increased DNA damage and mitochondrial dysfunction, older women are associated with increased oxidative stress, making them more prone to ovarian dysfunction and increased infertility.

## 5. Potential Dietary Interventions

As discussed earlier, diet is a risk factor for POI. Dietary supplementation of single compounds for POI has been reviewed extensively elsewhere [98,99,100]. In this section, we focus on some of the potential ways in which diet or dietary components can be beneficial in reducing the risk of POI.

### 5.1. Melatonin

Melatonin is a hormone produced by the pineal gland in the brain that regulates the sleep–wake cycle but is also a potent and versatile antioxidant supplement. Studies have shown melatonin reduces oxidative stress in a variety of organ systems [101]. However, less is known about the antioxidant properties of melatonin in the ovary and its link to human fertility. In women, melatonin has been found to improve oocyte quality and developmental competence and enhance the success of in vitro fertilisation (IVF) procedures [102]. There is now increasing evidence that melatonin has also been shown to protect against oxidative stress-induced damage in ovarian tissues and may protect against POI.

Melatonin is found in high concentrations in follicular fluid, with up to three times the concentration seen in serum, and melatonin increases in concentration as follicles grow [103,104]. Given that oxidative stress can contribute towards DNA damage in granulosa cells and the increasing concentrations of melatonin in growing follicles, the hormone has been suggested to play a pivotal role in ovarian health. Varying results have been observed in human studies with respect to melatonin and oxidative damage in follicles, with much of the research being done in animals. Melatonin supplementation has been shown to significantly reduce intrafollicular DNA damage while having a trend towards the lowering of lipid damage [102,105].

Melatonin has been used widely in artificial reproductive technologies (ART), and different studies have shown that it can increase follicular growth rate, improve oocyte quality, and increase fertilisation rate and the number of quality embryos, therefore leading to increased pregnancy outcomes [106]. Melatonin has been shown to improve oocyte maturation in animal and human studies [102]. A randomised control trial involving 85 IVF patients found melatonin supplementation (3 mg) significantly increases the number of mature oocytes [107]. Tamura et al. found melatonin supplementation significantly decreased the number of degenerate oocytes [102].

Melatonin has been shown in mice to enhance the repair of double-strand breaks via the non-homologous end joining (NHEJ) pathway to protect oocytes from the accumulation of DNA damage during prophase arrest [108], which helps prevent the deterioration of oocyte quality during meiotic maturation. This has an interesting parallel with POI, where one of the mechanisms of early loss of follicles is via the activation of DNA damage pathways. In addition, many genes found in familial POI are related to DNA damage repair, as highlighted previously, highlighting the importance of these pathways.

Interestingly, melatonin has also been shown to protect the ovaries from chemotherapy-induced damage, which may indicate an important role in protecting against early ovarian follicle loss. Barberino et al. (2017) performed melatonin pretreatment in mice prior to exposure to cyclophosphamide, and showed preserved ovarian function [109]. The results showed that melatonin reduced ROS levels in the ovary and increased mitochondrial activity, and alleviated oocyte loss. Other studies [110] have shown that melatonin prevents cyclophosphamide-induced over-activation of primordial follicles by inhibiting ovarian granulosa cell apoptosis and maintaining anti-Müllerian hormone (AMH) expression. The activated follicles undergo apoptosis, ultimately ending in POI. However, co-treatment with melatonin reduced primordial follicle activation and apoptosis of AMH-secreting granulosa cells in growing follicles. This highlights the cytoprotective effects of melatonin on the ovary and may provide new strategies to preserve the ovarian reserve against chemotherapy-induced damage. This approach to oncofertility is important for young cancer patients who undergo chemotherapy.

### 5.2. Mitochondria-Targeted Nutrient Therapy

Mitochondrial dysfunction resulting in oxidative stress is believed to have an important role in the risk of POI. Therefore, antioxidants may effectively counteract excessive ROS production particularly in women identified to have a mitochondrial-related gene mutation which can cause POI [7].

Several studies have reported on various antioxidant supplements and plant polyphenols in relation to mitochondrial function [111,112]. Epigallocatechin-3-gallate (EGCG) has been reported to improve mitochondrial function. In vitro studies point to EGCG reducing oxidative stress-induced apoptosis through the mitochondrial pathway in various cell lines [112]. In vivo, studies showed EGCG was capable of restoring respiration rates, reducing cell death via the mitochondrial pathway and upregulating mitochondrial antioxidant enzymes in several mammalian cells and tissues, including the heart and liver [112]. Additionally, in rat studies, EGCG had protective effects against mitochondrial dysfunction induced by cigarette smoke [113]. In relation to the ovaries, EGCG reduced chemotherapy-induced follicular atresia and activated the nuclear factor erythroid2-related factor 2 (Nrf2)/heme oxygenase-1 and superoxide dismutase 2 pathways in mice [114]. Additionally, EGCG was shown to promote the maturation of bovine oocytes in vitro [115] and diabetic oocytes in mice [116]. N-acetyl-l-cysteine is naturally found in plants and is a precursor to the amino acid L-cysteine and the antioxidant glutathione. N-acetyl-l-cysteine has been shown to increase mitochondrial functioning [117]. Consumption of nicotinamide mononucleotide (NMN) has been shown to improve oocyte quality in aged mice, and this was associated with mitochondrial function [118] and delays oocyte ageing [119,120]. Resveratrol was also shown to promote mitochondrial functioning and improves the maturation of oocytes from aged mice and humans in vitro [121]. Quercetin is a naturally derived flavonoid found in plants such as berries, broccoli, apples and onions [122,123]. Studies have shown that quercetin can prevent mitochondrial dysfunction by scavenging free radicals and stimulating antioxidant enzymes [123]. In vivo and in vitro studies have shown that quercetin can delay ovarian ageing and improve oocyte quality [123]. ATP production via OXPHOS involves electron transfer to ubiquinone, also known as CoQ10. CoQ10 has antioxidant properties and has been reported to decrease with ageing [34]. Supplementation with CoQ10 was shown to improve mitochondrial activity in oocytes of ageing female mice [124]. In a CoQ10 deficient PDSS-2 mouse model, where CoQ10 synthesis was interrupted in the mouse oocytes, the female ovaries contained no healthy follicles and resembled conditions seen in POI. CoQ10 supplementation prevented the loss of ovarian reserve and improved mitochondrial membrane potential and superoxide levels [124]. While there are a number of potential antioxidant compounds available, due to their limited bioavailability, the antioxidant effects of these compounds in the mitochondria are unclear.

More targeted antioxidants have been developed over the years, such as MitoQ and BGP-15. MitoQ is a ubiquinone moiety linked to a lipophilic cation which leads to the compound accumulating in the mitochondria [125]. Based on a systematic review of animal studies, MitoQ significantly reduces nitrotyrosine, a biomarker of protein oxidation and significantly increases mitochondrial membrane potential suggesting an improvement in mitochondrial function [125]. Another compound that accumulates in mitochondria is BGP-15 [126]. BGP-15 improves mitochondrial function and mtDNA copy number in oocytes from obese mice and improves oocyte quality [127]. While these compounds show an affinity for the mitochondria, it is unclear whether these compounds would reach the mitochondria in ovarian cells once consumed.

Human studies have found a reduction in oxidative stress after MitoQ consumption [128,129,130]. A study in older adults showed that consumption of 20 mg/d of MitoQ for 6 weeks reduced plasma-oxidised LDL (low-density lipoprotein), a marker of oxidative stress and improved age-related vascular function [128]. Studies in relation to exercise-induced oxidative stress showed chronic supplementation of 20 mg MitoQ per day was able to significantly reduce skeletal muscle H_2_O_2_ concentration [131] and postexercise plasma F2-Isoprostanes [132] oxidants as well as protected mitochondrial and nuclear DNA from damage [133]. However, these exercise studies were done on males only. Acute supplementation with MitoQ (80 mg) significantly increased plasma superoxide dismutase, an enzyme important in antioxidant defence, in patients with peripheral artery disease [134]. However, acute supplementation of 20 mg MitoQ did not have an impact on biomarkers of exercise-induced oxidative stress in men [133]. While these human studies suggest MitoQ supplementation reduces oxidative stress, no studies have looked at ovarian function.

### 5.3. Oily Fish

Fish and particularly oily fish is associated with a delay in natural menopause and improved ovarian health. In a large cohort study, it was found that a high intake of oily fish increased the onset age of menopause by 3.3 years [58]. Oily fish is a good source of anti-inflammatory omega-3, particularly the polyunsaturated fatty acids eicosapentaenoic acid (EPA) and docosahexaenoic acid (DHA). In one study with normal-weight women, supplementation with omega-3 for 1 month led to significantly lower inflammation and a significant reduction in FSH [135]. Furthermore, in mice studies, dietary treatment with omega-3 improved oocyte quality [136] and reversed high-fat-diet-induced ovarian decline [137]. Both these studies suggest a role for omega-3 in extending reproductive lifespan. Omega-3 has been shown to reduce oxidative stress-induced injury and DNA damage in human endothelial cells in vitro [138,139]. Furthermore, one study showed that EPA and DHA omega-3s significantly reduced intracellular ROS in endothelial cells [138]. While the beneficial effects of omega-3 are well known, fish oil supplements are sensitive to oxidation [140]. Therefore, combining it with other compounds, such as alpha-lipoic acid [141] or vitamin E [139], might help maintain its therapeutic efficacy. Furthermore, consuming fish instead of fish oil is likely to be more effective [142].

### 5.4. Dairy Consumption

Dairy food has been reported to improve reproductive function and delay natural menopause. From the Nurses’ Health Study II (NHSII) cohort, it was reported that low-fat dairy food intake, such as skim milk and yoghurt, may reduce the risk of early natural menopause (which the authors defined as natural menopause occurring before the age of 45 years) [143]. In another cohort study, women consuming more than 3 servings of low-fat dairy reached natural menopause 3.6 months later than those not consuming low-fat dairy products [144]. Similarly, a study that followed regularly menstruating women for 16 years found that a reduction in the rate of decline in anti-Mullerian hormone (AMH), an indicator of ovarian reserve, was associated with the consumption of dairy products, milk and fermented dairy products [145]. Furthermore, the study showed that lactose and free galactose intakes were also associated with a lower annual reduction in AMH [145]. This was further supported by a recent study showing high intake of galactose, and lactose was associated with the later onset of natural menopause in Japanese women [146].

Milk and dairy products are known to have antioxidant effects [147]. Milk and milk-containing products contain antioxidant compounds, including whey and casein proteins, vitamins A, C and E, and β-carotene. All subunits of casein were shown to have antioxidant capacity [148]. Additionally, some of the biopeptides produced from the digestion of casein have antioxidant properties [149]. The whey component of milk also possesses antioxidant properties, particularly as a source of cysteine amino acids which is a substrate in the synthesis of glutathione, an important antioxidant in the body [150]. Conjugated linoleic acid (CLA) is thought to be the most active antioxidant in milk fat and dairy products [150]. CLA has been shown to reduce lipid oxidation in vivo [151] and scavenges free radicals in vitro [152]. The well-known antioxidant properties of milk and dairy may have an important role in reducing damage and dysfunction in the ovaries and consequently reducing the risk of POI.

Dairy products have gut microbiota-modulating properties. Milk and milk-containing products are the main sources of “milk sugar” lactose which is hydrolysed into glucose and galactose. In vitro and animal studies report that lactose increases the growth of *Bifidobacterium* and *Lactobacillus* [153,154] and is considered a prebiotic [154]. *Lactobacillus* and *Bifidobacterium* can utilise and transform lactose into anti-inflammatory short-chain fatty acids, mainly acetate, propionate and butyrate [155]. Furthermore, fermentable dairy such as yoghurt has probiotic effects capable of retarding the growth of pathogenic bacterial strains in the gut, such as *Bacteroides fragilis* [156].

While dairy consumption has benefits, it is important to note that the accumulation of galactose can cause ovarian toxicity. Women with a condition called galactosemia lack the enzyme that metabolises galactose, known as galactose-1-phosphate uridyl transferase. Women who lack the enzyme or who have reduced activity of the enzyme tend to prematurely develop ovarian failure and menopause [157,158]. Additionally, high galactose consumption could promote menopause in the general public [159].

Taken together, the evidence suggests that consuming dairy could promote reproductive lifespan due to gut microbiota modulating and antioxidant effects. AMH has been favourably evaluated for use in POI diagnosis because it is a better marker of diminished ovarian reserve compared to FSH [160,161,162]. Since dairy has been shown to influence AMH levels in premenopausal women, it may suggest a role for dairy consumption in reducing the risk of POI. It will particularly be relevant for women who suffer from autoimmune disease or have a high BMI with gut microbiota disturbances. On the flip side, total dairy may contribute to increased fat intake, which could lead to increased oxidative stress and limit the beneficial effects of dairy. In line with this, cohort studies have associated low-fat dairy intake specifically as being associated with a delay in natural menopause. However, it is important to note that milk fat contains many antioxidant compounds, including lipophilic antioxidants (such as vitamin A and β carotene) and CLA. Therefore, the choice between consumption of full-fat or low-fat dairy may need to be taken into account in relation to other factors, including BMI. Furthermore, caution needs to be taken when consuming a high intake of dairy in relation to the accumulation of galactose. Additionally, dairy consumption may not be suitable for women with lactose intolerance or galactosemia.

### 5.5. Vitamin C and E

Ascorbic acid (AA), or water-soluble vitamin C, is a well-known natural antioxidant (reviewed extensively elsewhere). Supplementation with AA was shown to increase progesterone and pregnancy success in women with luteal phase defects suggesting a role during heavy exercise-induced disturbances to ovulation [163]. In ageing female mice, AA was able to prevent the effects of ovarian ageing, namely reduction of ovarian volume, number of ovarian follicles and granulosa cells [164]. Vitamin C supplementation may also have a role in reducing the oxidative damage caused by electromagnetic radiation (EMR) [165]. Oral administration of vitamin C in rats significantly reduced oxidative stress-induced apoptosis after rats were exposed to EMR [165]. However, in the same study, vitamin C also reduced serum AMH.

Vitamin C has promising effects in relation to POI. Vitamin C is capable of reducing exercise-induced oxidative stress and psychological stress in women, which are both risk factors for POI [80]. In mice, transplanting human amniotic epithelial cells treated with vitamin C in the ovaries of a mouse model of PI, improved ovarian function [166].

Vitamin E is a group of compounds that includes tocopherols, with α-tocopherol being the main form absorbed in humans. α-tocopherol is well known for its antioxidant effects in ovarian tissue and follicular fluid [167]. Additionally, α-tocopherol is significantly lower in women with POI than in women with normal menstrual cycles [168].

## 6. Final Remarks

Personalised lifestyle changes particularly based on the genetic background of women with POI hold great potential. Currently, genes associated with POI seem to fall into a number of key biological processes, such as folliculogenesis, meiosis/DNA damage repair, energy and metabolism and sex steroid metabolism. By understanding the genetic background, specific lifestyle changes, such as diet and exercise recommendations, can be tailored to their unique needs, helping to delay the development of POI or mitigate the symptoms and manage the condition more effectively. While there may be a complex and overlapping pattern of mechanisms related to genetic background, lifestyle changes that reduce oxidative stress and inflammation and address specific nutrient deficiencies may provide better outcomes for women with POI, and perhaps delay the age of onset of those genetically predisposed, as we have seen for those at the natural age of menopause and those with chemically or surgically induced menopause.
